# Thermoelectric Efficiency of Silicon–Germanium Alloys in Finite-Time Thermodynamics

**DOI:** 10.3390/e22101116

**Published:** 2020-10-02

**Authors:** Patrizia Rogolino, Vito Antonio Cimmelli

**Affiliations:** 1 Department of Mathematical and Computer Sciences, Physical Sciences and Earth Sciences, University of Messina, Viale F. Stagno d’Alcontres, 31, 98166 Messina, Italy; progolino@unime.it; 2 Department of Mathematics, Computer Science and Economics, University of Basilicata, Viale dell’Ateneo Lucano, 10, 85100 Potenza, Italy

**Keywords:** finite-time thermodynamics, Silicon–Germanium alloys, minimum of thermal conductivity, efficiency of thermoelectric systems, minimal energy dissipation

## Abstract

We analyze the efficiency in terms of a thermoelectric system of a one-dimensional Silicon–Germanium alloy. The dependency of thermal conductivity on the stoichiometry is pointed out, and the best fit of the experimental data is determined by a nonlinear regression method (NLRM). The thermoelectric efficiency of that system as function of the composition and of the effective temperature gradient is calculated as well. For three different temperatures (T=300 K, T=400 K, T=500 K), we determine the values of composition and thermal conductivity corresponding to the optimal thermoelectric energy conversion. The relationship of our approach with Finite-Time Thermodynamics is pointed out.

## 1. Introduction

In recent years, Silicon–Germanium (SiGe) alloys have become very important in technology, since some of their properties such as, for example, their efficiency in energy conversion, may be improved by adjusting their stoichiometry. Indeed, alloys of the type $Si_{c}Ge_{1-c}$, where c∈[0,1] is a stoichiometric parameter which varies along a direction *z* in the system, are widely used in energy production and management [1,2,3,4,5,6]. The thermoelectric efficiency is defined as $\eta_{el}=\frac{P_{\mathrm{el}}}{{\dot{Q}}_{\mathrm{tot}}}$, with $P_{\mathrm{el}}$ the obtained electric power and ${\dot{Q}}_{\mathrm{tot}}$ the heat per unitary time entering the system, [7,8,9]. It can be proven that $\eta_{el}$ is an increasing function of the material function ZT, where *T* is the absolute temperature while the figure-of-merit *Z* is given by $Z=\frac{\epsilon^{2}\sigma_{e}}{\lambda }$, where ϵ is the Seebeck coefficient, $\sigma_{e}$ the electrical conductivity, and λ is the thermal conductivity of the material [9]. Then in the literature one can find several methods to enhance *Z* [10,11,12]. One of the most successful strategies is the use of nonlinear nanomaterials [13,14,15], namely nanomaterials in which some nonlinear transport equations hold. Indeed, for those materials an external control of the flux of heat carriers is possible, leading to a reduction of the thermal conductivity, and hence to an increment of *Z* [16,17,18,19]. The efficiency of a homogeneous thermoelectric system has been calculated in [20,21], wherein some strategies to enhance its performance have been pointed out. The thermoelectric efficiency of a nanosystem of variable composition has been studied in several papers by the present authors [22,23,24]. In particular, in [23], we have obtained the analytical representation of the thermal conductivity of a nanowire as function of its composition *c*, as the sum of two exponentials, each depending on 3 parameters, whose value was determined by the experimental data through NLRM.

On the other hand, in several cases the constituents of thermoelectric energy generators can have macroscopic dimension, so that the thermoelectric efficiency of graded systems at macroscale needs to be investigated as well. In such a framework, the possibility of application of the nonlinear model used in [22,23,24] needs to be tested, since at macroscopic scale nonlinear effects are less evident. Thus, in this paper we explore the possible extension of the model at macroscopic scale, by considering a SiGe graded wire, of length L=3 mm. We investigate the dependence of its performance as thermoelectric energy generator as function of the composition and of the effective temperature gradient applied to its boundaries, and determine the conditions under which such an efficiency is maximum. Our conclusion will be that the model still leads to previsions which are physically sound and acceptable for the system at hand, although they differ from those obtained in [23].

Moreover, as additional result of the present research, going a step further with respect to [23], we also improve the constitutive equation of the thermal conductivity, which now depends by only 4 parameters instead of the 6 parameters used in [23]. Such an improvement is not easy, as it could appear at a first look, because a reduction of the number of free parameters in general increases the numerical error which affects the fit, and the new fit can be used in the applications only if the error on it is kept at an acceptable level. In Section 2 we discuss this problem and show that our fit reproduces accurately the experimental data.

Then, we calculate the heat conductivity at T=300 K,T=400 K, and T=500 K, corresponding to the experimental data at our disposal, and prove that for each temperature there is only one value of *c* in the interval [0,1] which minimizes the local rate of entropy production, i.e., which corresponds to the optimal efficiency of the thermoelectric energy production.

The article has the following structure.

In Section 2, we apply a NLRM to obtain the best fit of the curve which represents the thermal conductivity of a wire of length L=3 mm as function of its composition *c*.

In Section 3, we first give a sketch of the nonlinear model we are facing with, and calculate the form of the local rate of entropy produced along the thermoelectric process. Then, under the assumption that the optimal efficiency is achieved in correspondence of a minimum of the local rate of entropy produced, we determine the theoretical expressions of *c* which minimize such a rate.

In Section 4, we calculate the effective values of *c* given by the theoretical expressions found in Section 3 and discuss this result taking into account the characteristic properties of nanosystems.

Finally, in Section 5, we interpret the present approach within the frame of Finite-Time Thermodynamics [25,26,27]. In particular, we show that our assumption of minimum entropy production can be considered a consequence of a global variational principle which is suitable for application in Finite-Time Thermodynamics.

## 2. Constitutive Equation of Thermal Conductivity

In this section, by using MATHLAB (http://www.mathlab.mtu.edu/mediawiki/index.php/Main_Page), we apply a NLRM [28,29] to determine the best fit of the experimental data for the heat conductivity of a $Si_{c}Ge_{1-c}$ wire of length L=3 mm as function of its composition parameter c∈[0,1].

In Ref. [23], starting from the experimental data in [30,31,32], such a procedure led us to the following constitutive equation

(1) $$\lambda \left(c\right)=A^{\prime }e^{B^{\prime }c^{2}+D^{\prime }c}+E^{\prime }e^{F^{\prime }c^{2}+G^{\prime }c}$$

with $A^{\prime }$, $B^{\prime }$, $D^{\prime }$, $E^{\prime }$, $F^{\prime }$ and $G^{\prime }$ as material parameters, determined by a NLRM. The constitutive Equation (1) led us to a very good accordance with the experimental data [23].

In the present paper, we go a step further, starting from the observation that since we have pure Ge for c=0, and pure Si for c=1, for Equation (1) the following constraints on $A^{\prime }$, $B^{\prime }$, $D^{\prime }$, $E^{\prime }$, $F^{\prime }$ and $G^{\prime }$ must be satisfied for any value of the temperature *T*

(2) $$\lambda \left(0\right)=A^{\prime }+E^{\prime }=\lambda_{Ge}\;\;\;\;\;\;\;\;\lambda \left(1\right)=A^{\prime }e^{B^{\prime }+D^{\prime }}+E^{\prime }e^{F^{\prime }+G^{\prime }}=\lambda_{Si}$$

where $\lambda_{Ge}$ and $\lambda_{Si}$ are the thermal conductivity of pure Ge and pure Si, respectively. As a consequence, only 4 independent parameters are necessary, once the experimental values of $\lambda_{Ge}$ and $\lambda_{Si}$ at a fixed temperature are known. To obtain a manageable 4-parameters representation is not an easy task, because, in general, the smaller the number of free parameters in the fitting function, the higher the numerical error affecting the fit. In the present paper, we determine a new fit, with 4 independent parameters only, which is reliable and introduces a small error. For the new 4-parameters representation, the analysis of the data in [30,31,32] suggests us to look for a best-fit curve of the form

(3) $$\lambda \left(c\right)=f\left(A,B,D,E\right)e^{Ac^{2}+Bc}+g\left(A,B,D,E\right)e^{Dc^{2}+Ec}$$

where *A*, *B*, *D*, *E*, are the unknown parameters to be determined by NLRM and f(A,B,D,E) and g(A,B,D,E) are suitable parameters-dependent coefficients. Finally, the conditions $\lambda \left(0\right)=\lambda_{Ge}$ and $\lambda \left(1\right)=\lambda_{Si}$ give the following expressions of the functions f(A,B,D,E) and g(A,B,D,E)

$$\begin{array}{c} f\left(A,B,D,E\right)=\frac{\lambda_{Si}-\lambda_{Ge}\;e^{D+E}}{e^{A+B}-e^{D+E}},\;\;\;g\left(A,B,D,E\right)=\frac{-\lambda_{Si}+\lambda_{Ge}\;e^{D+E}}{e^{A+B}-e^{D+E}} \end{array}$$

Thus, our fitting curve can be obtained once the parameters *A*, *B*, *D*, and *E* are determined. In order to calculate them, first we estimate some initial values for parameter entering Equation (3). Then, in the set of the possible couples (c,λ(c)), i.e., in the strip $\left\{\left[0,1\right]\times \left[0,\infty \right]\right\}\subset R^{2}$, we generate the curve determined by the estimated values of the parameters, and adapt them in such a way that the Euclidean distance in $R^{2}$ between the fitting curve and the experimental points is as small as possible. Let’s notice that the total error affecting the fit, i.e., the sum of the squared distances between the experimental points and the fitting curve, is of the same order of magnitude for all the temperatures considered here.

**Figure 1:**
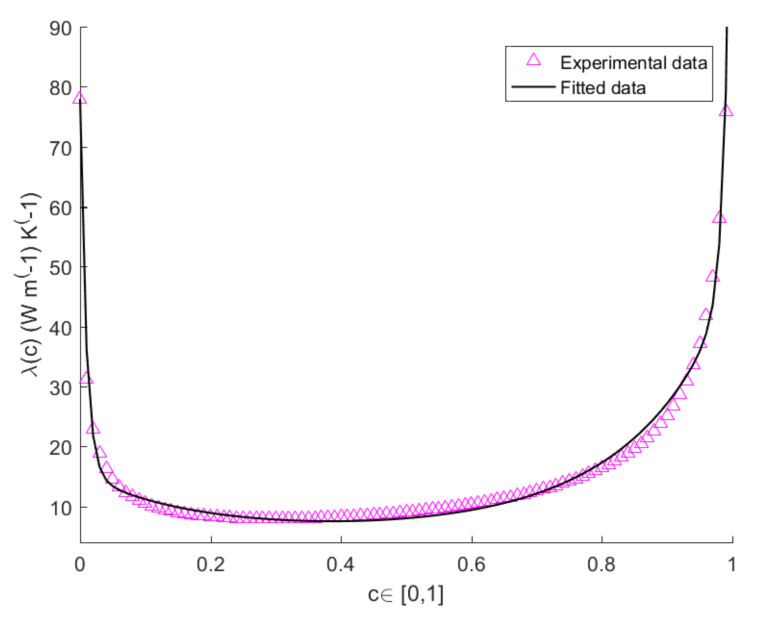
Plots of the calculated vs. measured values of thermal conductivity of a $Si_{c}Ge_{1-c}$ wire of length L=3 mm as function of *c*, at temperature T=300 K.

**Figure 2:**
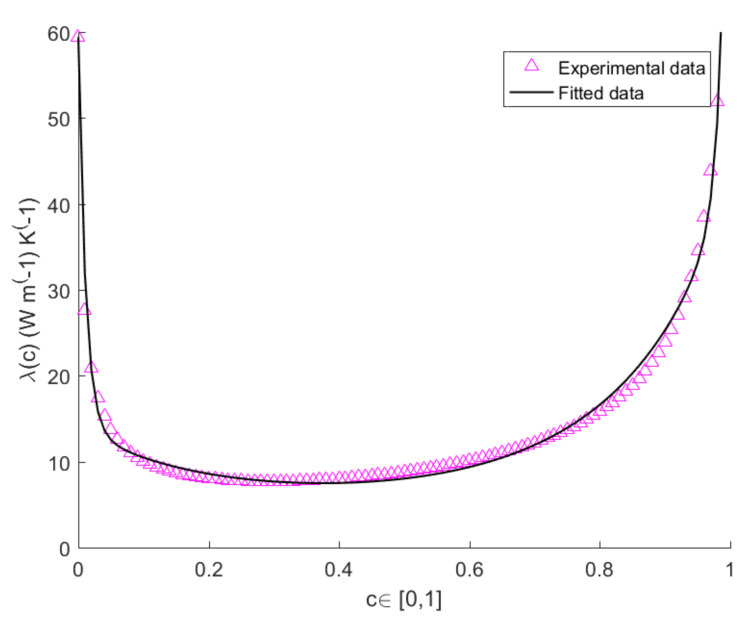
Plots of the calculated vs. measured values of thermal conductivity of a $Si_{c}Ge_{1-c}$ wire of length L=3 mm as function of *c*, at temperature T=400 K.

**Figure 3:**
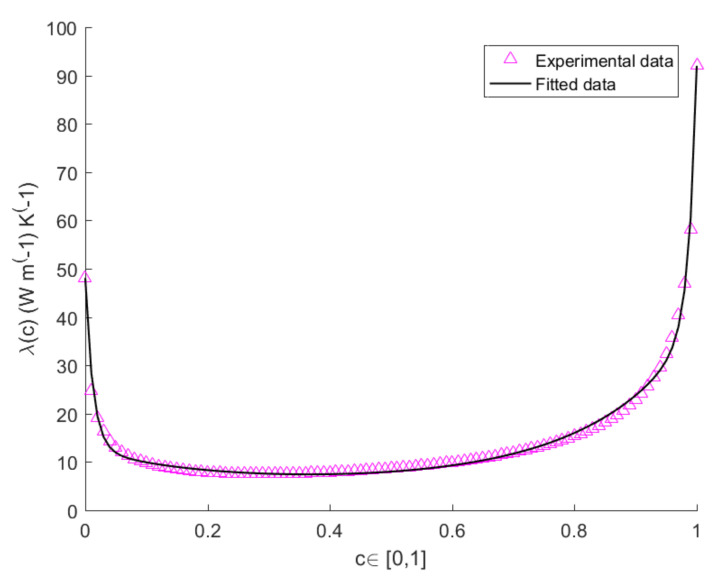
Plots of the calculated vs. measured values of thermal conductivity of a $Si_{c}Ge_{1-c}$ wire of length L=3 mm as function of *c*, at temperature T=500 K.

The values of *A*, *B*, *D*, *E* at T=300 K,T=400 K,T=500 K are shown in Table 1 for L=3 mm. The values of the heat-conduction parameter for bulk systems of pure Si and pure Ge at T=300 K, T=400 K and T=500 K, are shown in Table 2. The plots in Figure 1, Figure 2 and Figure 3 show the measured and theoretical values of λ(c) expressed by Equation (3), at T=300 K, T=400 K and T=500 K, for L=3 mm. By comparing the pink and black curves in Figure 1, Figure 2 and Figure 3 we argue that fitting curve reproduces accurately the experimental data. It is evident from the figure the presence of two narrow zones, close to the extremes of the interval [0,1], in which λ varies steeply, while it remains almost constant in the other points of the interval. The variation of λ with *c* is more evident in the subintervals [0,0.1] and [0.9,1] because a small quantity of impurities is capable of enhancing the phonon scattering, and, as a consequence, to reduce very much heat conductivity with respect to the one of the pure system.

## 3. Best Efficiency in Thermoelectric Energy Conversion

The system analyzed here is a graded $Si_{c}Ge_{1-c}$ wire of length *L* crossed by an electric current i, on which acts an electric field E. The right-hand side (z=L), is kept at the hot temperature $T_{h}$, while the left-hand side, (z=0), is kept at the cold temperature $T_{c}$. Since the material composition changes with position, at right-hand side (z=L) we have only Silicon while at left-hand side, (z=0) we have only Germanium. Then, the system is similar to a junction of different materials at the ends of which is applied a difference of temperature $T_{h}-T_{c}$. As is well known, such a type of junction is capable of generating a difference of electric potential at its ends, and this phenomenon is the classical thermoelectric effect [16,17]. As a consequence of the generation of this difference of potential, there are an electric current i flowing uniformly inside the system from left to right, and an electric field E acting on the system. The difference of temperature at the ends of the wire is generated by given amount of heat per unit time ${\dot{Q}}_{\mathrm{tot}}$ which enters uniformly into the hot side of the element.

The model is represented by:

- The local energy balance [21]
  (4) $$\rho \frac{du}{dt}+\nabla \cdot q=E\cdot i\;$$
  where ρ is the mass density, *u* the specific internal energy and q the heat flux;
- the constitutive equation for the heat flux
  (5) q=−∇q·l−λ(1−b)∇T+Πi
  where l denotes a characteristic-length vector, proportional to the heat flux q, *b* is a dimensionless quantity smaller than 1 and depending on $q^{2}$, and Π is the Peltier coefficient [9,17];
- the constitutive equation for the electric current
  (6) $$i=-\sigma_{e}\epsilon \nabla T+\sigma_{e}E$$

Here the Peltier and Seebeck coefficients Π and ϵ, as well as the electric conductivity $\sigma_{e}$, are supposed to be constant. It is worth noticing that for the nonlinear heat conductor presented here the classical second Kelvin relationship Π=ϵT, which holds in Linear Irreversible Thermodynamics [9,17], in general, is no longer true (see [21] for a detailed discussion of this point). In our analysis we assume also that the thermal conductivity λ(c,T) can be approximated with its expression at the hottest side, namely $\lambda (c,T_{h})$.

From the physical point of view, the previous hypotheses mean that we restrict our investigation to rigid conductors whose thermal and electric functions have small variations with respect to temperature, and the variation of λ with the composition is preeminent in influencing the thermoelectric behavior. Of course, this is not the most general case, and the present investigation must be considered only as a first step toward a complete analysis of the thermoelectric behavior of composition graded materials.

From the mathematical point of view, as it will be shown below, the previous hypotheses lead to a problem of determination of the points of minimum of a function of two independent variables. In such a case, some conditions ensuring that such minima always exist can be determined. On the other hand, if the material functions would depend on the temperature too, the same problem should be considered for a function of three independent variables, as in Ref. [24]. In such a case, it is much more difficult to determine the conditions which ensure the existence of minima. Moreover, more data on the dependence of the material functions on temperature are necessary. Indeed, currently we are considering such a problem, and the results will be included in a forthcoming article.

Under the conditions discussed above, if we further assume that both q and E depend only on the position on the longitudinal axis *z*, and that q and i are parallel, by some lengthy calculations we obtain that the local rate of energy dissipated along a thermoelectric process is [23]

(7) $$E=\frac{i^{2}}{\sigma_{e}}+i\left[\epsilon \;T_{h}-\left(\Pi -\bar{E}\;\;\bar{l}\right)\right]\nabla T+\lambda \left(1-b\right){\left(\nabla T\right)}^{2}$$

where $\bar{E}$, and $\bar{l}$ denote the mean values of |E|, and |l| on the interval [0,L], respectively. In what follows we restrict ourselves to steady-state situations, which are usual for thermoelectric converters, and exploit Equation (7) in order to determine the situation in which the efficiency as thermoelectric energy converter of the system under consideration is maximum. Indeed, our main assumption is that the optimal efficiency is achieved in correspondence of a minimum of the rate of energy dissipated. Such a hypothesis lies on the observation that the efficiency is reduced by dissipative effects induced by the heat and electric transport. In Section 4 we will discuss it within the framework of Finite-Time Thermodynamics [25,26,27].

Equation (7) provides an expression of the local rate of energy dissipated, depending on the temperature gradient and on $T_{h}$ and *c* through the thermal conductivity $\lambda (T_{h},c)$. Thus, if $T_{h}$ is fixed at one of the constant values T=300 K,T=400 K,T=500 K, function E depends only on the gradient of temperature, and on the composition *c*. Furthermore, the hypothesis that the temperature gradient is parallel to *z* allows the further approximation $\nabla T=\frac{dT}{dz}≃\frac{T_{h}-T_{c}}{L}$. Then, Equation (7) rewrites as follows

(8) $$E(c,x)=\frac{i^{2}}{\sigma_{e}}+i\left[\epsilon \;T_{h}-\left(\Pi -\bar{E}\;\;\bar{l}\right)\right]x^{2}+\lambda \left(c\right)\left(1-b\right)x^{4}$$

where

(9) $$x\equiv \sqrt{\frac{T_{h}-T_{c}}{L}}$$

In the following we look for the possible minima of function $E\left(c,x\right)$. It is easily proved that those points, which will be denoted by $\left(c_{s},x_{s}\right)$, are the stationary points $c_{s}$ of λ(c), and the values

(10) $$x_{s}=\sqrt{\frac{-i\;[\epsilon \;T_{h}-\left(\Pi -\bar{E}\;\;\bar{l}\right)]}{4\;\lambda_{s}\left(1-b\right)}}$$

with $\lambda_{s}\equiv \lambda \left(c_{s}\right)$. They exist if the inequality

(11) $$i\;[\epsilon \;T_{h}-\left(\Pi -\bar{E}\;\;\bar{l}\right)]<0$$

holds. The relationship (11) can be considered as a unilateral constraint on the physical parameters which characterize the model.

By the analysis of the Hessian matrix of the function $E\left(c,x\right)$, it follows that the condition that must be fulfilled for the existence of a minimum for it is that the thermal conductivity has a minimum in $c_{s}$, and that the further constraint

(12) $$2i\left[\epsilon \;T_{h}-\left(\Pi -\bar{E}\;\bar{l}\right)\right]+12\lambda_{s}x_{s}^{2}\left(1-b\right)>0$$

is satisfied. Although the first addendum in the left-hand side of Equation (12) is negative because of the constraint (11), the second one is positive, and hence the inequalities (11) and (12) can be satisfied contemporarily. Thus, the points of minimum for E(c,x) exist. From now on we denote by $\left(c_{opt},x_{opt}\right)$ such points.

## 4. Results

In this section, we discuss the properties of the minima $\left(c_{opt},x_{opt}\right)$ of E(c,x), calculated by using MATHEMATICA (https://www.wolfram.com/mathematica/).

At T=300 K, E(c,x) attains a minimum at c=0.385989. In this point $\lambda =7.51235\;\;Wm^{-1}K^{-1}$.

At T=400 K, E(c,x) attains a minimum at c=0.375079. In this point $\lambda =7.48291\;\;Wm^{-1}K^{-1}$.

At T=500 K, E(c,x) attains a minimum at c=0.36537. In this point $\lambda =7.42273\;\;\;Wm^{-1}K^{-1}$.

The previous results are summarized in Table 3, wherein $\lambda_{opt}\equiv \lambda \left(c_{opt}\right)$.

Let us now compare the present results with those obtained in [23]. In both cases, there are no local minima of λ in the zones where λ is more steep, namely the optimal efficiency takes place in the zone where λ is almost constant. The values of $c_{opt}$ are a little bit smaller (from 0.36 to 0.38) with respect to the values found in [23] (from 0.44 to 0.41). However, in both cases we got three points of minimum very close each other, which correspond to small differences in λ. Meantime, the values of $\lambda_{opt}$ obtained here for a wire of length L=3 mm are almost an order of magnitude higher with respect to those obtained in [23] for L=100 nm, and comparable with (but smaller than) those obtained for L=30 nm. It is worth noting that in [23] we obtained a marked difference between the values of $\lambda_{opt}$ for L=30 nm and those for L=100 nm. It can be considered a size effect, i.e., a strong dependency of the material properties on the dimension of the system. This is just what we observed in [23], since for L=30 nm we obtained $\lambda_{opt}$ of the order of magnitude of 30 W m${}^{-1}$ K${}^{-1}$, while for L=100 nm we obtained $\lambda_{opt}$ of the order of magnitude of 0.4 W m${}^{-1}$ K${}^{-1}$. Size effects are very frequent in heat conduction in nanosystems, and manifest themselves when the physical dimension of the heat conductor becomes comparable with, or smaller than, the mean free path of the heat carriers [16,17]. These effects disappear at macroscopic length-scale. To verify such a property in our case, the dependency of $\lambda_{opt}$ on the size of the system at macroscopic scale deserves further investigation. For such a study we need new experimental data, for length smaller and higher than 3 mm. Currently we are not aware of such data, but we are looking for them in the literature.

## 5. Relation with Finite-Time Thermodynamics

The early studies on the efficiency of thermodynamic engines were based on the concept of Carnot cycle, which means a quasi-static, i.e., reversible, thermodynamic cycle constituted by two isothermal and two adiabatic arcs in the state space, in which a thermodynamic system adsorbs, at constant temperature, a quantity of heat $Q_{H}$ by a hottest source at temperature $T_{H}$ and gives, at constant temperature, a quantity of heat $Q_{C}$ to a cold reservoir at temperature $T_{C}$. Along the cycle, the system produces a net amount of work $W=Q_{H}-Q_{C}$. The efficiency of this cycle is $\eta \equiv W/Q_{H}=\left(Q_{H}-Q_{C}\right)/Q_{H}=1-Q_{C}/Q_{H}$. Carnot was the first to prove that such efficiency takes the form $\eta_{C}=1-T_{C}/T_{H}$. Since a quasi-static transformation requires an infinite time, the Carnot efficiency $\eta_{C}$ is not suitable to describe the efficiency of real processes, which take over in a finite time. For those processes it is more useful to calculate the efficiency as the ratio $\eta =P_{\mathrm{ex}}/{\dot{Q}}_{\mathrm{tot}}$, where $P_{\mathrm{ex}}$ is the extracted work per unitary time and ${\dot{Q}}_{\mathrm{tot}}$ is the heat supplied to the system per unitary time. A simple model of system operating in finite time is provided by the Curzon-Ahlborn endoreversible engine [33]. For that system the efficiency at maximum power, i.e., when the system extracts the maximum power, can be proved to be $\eta_{CA}=1-\sqrt{T_{C}/T_{H}}$, so that $\eta_{CA}<\eta_{C}$. Thus, for real processes, the central question is to investigate how much the efficiency deteriorates when the cycle is operated in a finite time. This is the task of Finite-Time Thermodynamics, a modern nonequilibrium theory, which has been developed in the last four decades by Andresen, Salamon, Stephen Berry et al. [25,26,27,34].

Classical thermoelectricity can be considered to lay within the frame of Finite-Time Thermodynamics for the following reasons:

- The definition of the thermoelectric efficiency as $\eta_{el}=P_{\mathrm{el}}/{\dot{Q}}_{\mathrm{tot}}$ (see Section 1) does not require quasi-static transformations along an infinite time. Indeed, if one remains in the frame of linear thermodynamics, i.e., with linear constitutive equations for heat flux and electrical current, it can be proved that the maximum efficiency is [9]
  (13) $$\eta_{max}=\eta_{C}\frac{1-1/\xi }{1+1/\xi }$$
  wherein $\xi \equiv \sqrt{ZT+1}$. Hence, $\eta_{max}<\eta_{C}$, in accordance with the tenets of Finite- Time Thermodynamics.
- In the situation considered in the present investigation, two time scales appear: the scale of the electric effects, and that of the thermal ones. Indeed, according to the general tenets of Extended Irreversible Thermodynamics, the constitutive Equations (5) and (6) can be obtained by the following balance laws for the heat flux and for the electric-charge flux, namely the electric current i [16,17]:
  (14) $$\tau_{q}\dot{q}+q=-\nabla q\cdot l-\lambda (1-b)\nabla T+\Pi i$$
  (15) $$\tau_{i}\dot{i}+i=-\sigma_{e}\epsilon \nabla T+\sigma_{e}E$$
  where $\tau_{q}$ is the relaxation time of the heat flux and $\tau_{i}$ the relaxation time of the electric current. On the other hand, at the macroscopic length-scale electric phenomena are faster of the thermal ones, so that the electric relaxation time is much shorter that the thermal one. Thus, the condition $\tau_{i}<<\tau_{q}$ allows the regarding of the thermal evolution as a finite-time process with respect to the electric one.

Equation (13) implies that the higher ξ, the higher $\eta_{max}$, so that several researches in recent decades focused on the methods to enhance ξ, i.e., to enhance ZT. However, this is not an easy task. For instance, still remaining in a linear theory, it can be easily proved that for the wire considered here, ${\dot{Q}}_{\mathrm{tot}}=\lambda \left(T_{h}-T_{c}\right)/L+\Pi i$ [21], so that for fixed $T_{h}-T_{c}$ and *i*, a reduction of ${\dot{Q}}_{\mathrm{tot}}$ can be realized, from the technical point of view, only by a reduction of λ, i.e., by an increment of $Z=\epsilon^{2}\sigma_{e}/\lambda$, producing so an enhancement of the efficiency. However, a reduction of λ is connected with an increment of the phonon scattering inside the thermoelectric solid [16,17], and this produces dissipation which, in turn, reduces $P_{\mathrm{el}}$. Thus, numerator and denominator cannot be controlled independently in the expression of $\eta_{el}$. On the other hand, to optimize only one of them is not sufficient to obtain the best efficiency, as argued by Hoffmann in [34], where it is shown by a meaningful example that the maximum power does not correspond to the minimum dissipation, and hence to the best efficiency.

In the present investigation we propose a new procedure in the realm of Irreversible Thermodynamics (Classical and Extended) which is capable of overcoming the difficulties mentioned above, since it does not focus on the power output but on the energy dissipated along the thermoelectric process. We proceed as follows. We disregard all the losses related to the production of ${\dot{Q}}_{\mathrm{tot}}$ and to the management of the generated difference of electrical potential, and we focus only on the thermodynamic process inside the thermoelectric wire. As illustrated in Section 3, it consists of the generation of an electric potential after that an amount of heat per unit time ${\dot{Q}}_{\mathrm{tot}}$ entered uniformly inside the system. Such a heat flow produces dissipation by Joule effect, which, in any point *z* of the system and at any time *t*, is given by the rate of energy dissipated E(c(z),x(z,t)) calculated in Equation (8). Please note that since we suppose the absence of any mechanical friction, the sole dissipation of energy is due to the Joule effect. Then, we argue that the smaller the energy dissipated by Joule effect, the higher the efficiency in the process of thermoelectric energy conversion. It is worth remarking that E(c(z),x(z,t)) is a local quantity, so that our hypothesis of minimum energy dissipated is local.

At this point one may wonder if our point of view can follow by a global variational principle which holds for a wider class of thermoelectric systems. To investigate such a possibility, let’s consider a thermoelectric system, and let Σ its state space, spanned by a set of *n* thermodynamic variables $X_{1},\;\ldots ,\;X_{n}$. Moreover, let $A\equiv (X_{1A},\;\ldots ,\;X_{nA})$ and $B\equiv (X_{1B},\;\ldots ,\;X_{nB})$ denote two generic points of Σ. The following statement can be expected to hold.

Principle of Minimum Energy Dissipated

*Let S a thermoelectric system undergoing a thermodynamic process of conversion of a given amount of heat per unit time into an electric-power output, and let such a process represented by a regular curve between two fixed thermodynamic states A and B of the system. Then, among all the possible processes represented by a curve of extremes A and B in Σ, the most efficient one is the process in which the total energy dissipated by Joule effect achieves a minimum.*

If a thermoelectric process is represented by a regular curve γ in the state space, the total energy dissipated is given by

(16) $$E_{tot}=\int_{\gamma }E\left(c,\;x\right)dl$$

where dl denotes the infinitesimal arc-length of γ.

The Principle of minimum energy dissipated states that the most efficient thermoelectric process is the one in which $E_{tot}$ is minimum.

For the one-dimensional system considered here, the previous principle implies that the best efficiency is obtained when the local energy dissipated per unit time (i.e., the local power dissipated) E(c,x) is minimum. To prove that, we first observe that at the constant temperature $T_{h}$, the constitutive Equations (5) and (6) depend only on the composition *c* and on the temperature gradient ∇T, which we have approximated by the effective gradient *x*. Thus, the sole thermodynamic variables are the couple (c,x). Moreover, we are considering the steady-state situation, in which a process of thermoelectric energy conversion takes place with the system in a constant state. In such a case the different curves reduce to different points of Σ, and among them, we are looking for the state for which the energy dissipated is minimum. In such a situation the total energy dissipated in any point is given by

(17) $$E_{tot}=\int_{0}^{L}\int_{0}^{\tau }\bar{E}\left(z,\;t\right))dzdt$$

where τ is the duration of the thermodynamic process, and the superposed bar means that E(c(z),x(z,t)) was explicated as function of *z* and *t*.

On the other hand, being $\bar{E}\left(z,\;t\right))$ a positive quantity, the right-hand side of Equation (17) is minimum if, and only if, the integrand function is minimum in any point of the domain of integration, or, equivalently, if, and only if, E(c,x) is evaluated in the state $\left(c_{opt},x_{opt}\right)$ found in Section 3 and Section 4.

In the technical applications the previous result allows the determination a priori of the part of the conductor where the energy conversion is optimal by modulating the dependence of *c* on *z*.

This proves that for the system considered in the present investigation, the global and local forms of the principle are equivalent. Of course, this is a very particular case, and it is important to underline that the previous conclusions are no longer true for different systems and in non-stationary situations. In future research, we plan to extend such investigation to more general systems, for instance, to deformable graded continua, in order to verify the possible extensions of the Principle of minimum energy dissipated.

It is important to note the different physical dimensions of E and $E_{tot}$: the first one is a power density, measured in $Jm^{-1}s^{-1}$, the second one is the total energy dissipated along the process, which is measured in *J*. We observe that the local form of our principle requires that the power density E(c,x) takes a minimum, the global form, instead, requires that the total energy $E_{tot}$ takes a minimum.

**Remark** **1.** *The results above are in accordance with Gyarmati approach to Irreversible Thermodynamics. According to this approach, the fundamental laws of the thermodynamics of dissipative processes can be resumed into a very general variational principle, formulated by Gyarmati both in local and global forms [35]. Such a principle allows several particular formulations, and it is very useful in optimization problems, as those which are typical of Finite-Time Thermodynamics. For a general analysis of the principle we refer the reader to the paper [36]. The investigation of a possible formulation of the general tenets of Finite-Time Thermodynamics in view of the Gyarmati variational principle for dissipative processes constitutes a very interesting field of investigation, which, however, is beyond the scope of the present article.*

It is worth noticing that the Principle of minimum energy dissipated is only one of the possible criteria for the optimization of the performance of finite-time heat engines. For instance, some authors proposed the maximization of the power output, some others the maximization of the ratio between the power output and the heat adsorbed, i.e., the quantity $\eta =\frac{P_{\mathrm{out}}}{{\dot{Q}}_{\mathrm{tot}}}$, and others the minimization of the entropy production (see [25] for a discussion of this topic). Each of those approaches presents vantages and disadvantages. For instance, the power maximization aims at designing highly performant heat engines, the maximization of the η aims at obtaining good performance with acceptable costs in energy, the minimization of entropy production, or, equivalently, of energy dissipated, aims at preserving the natural resources. Regarding this aspect, in the literature one can also find an ecological criterion which requires maximization of the difference between the power output and the energy dissipated [37]. Such a criterion seems to be a good compromise between power enhancement and acceptable entropy production.

The analysis of the efficiency of thermoelectric graded systems in view of the different criteria illustrated above offers interesting perspectives for future research.

At the very end, we underline again that the previous analysis regards only the process of thermoelectric energy conversion inside the wire, while the dissipation inside the surrounding is neglected. Of course, this working hypothesis is only an approximation since, in general, is not easy to separate the entropy production of the surrounding from that of the heat conductor. Hence, a complete analysis of thermoelectric energy conversion should take into account the dissipation due to the production of ${\dot{Q}}_{\mathrm{tot}}$ and that due to the transport and management of the obtained difference of electric potential. However, such a study is outside the scopes of the present research, and is more pertinent to the field of Engineering. The previous considerations serve only to explain why the problem investigated here, especially the procedure carried out in Section 2 and Section 3, can be considered to be typical of Finite-Time Thermodynamics.

## Figures and Tables

**Table 1:** Values of *A*, *B*, *D*, *E* in Equation (3) for a $Si_{c}Ge_{1-c}$ wire of length L=3 mm.

Temperature	A	B	D	E
T=300 K	4.8706	−3.76	109.452	−108.953
T=400 K	91.804	−91.351	4.416	−3.3127
T=500 K	4.0667	−2.9717	80.4998	−80.0781

**Table 2:** Thermal conductivity in (W m${}^{-1}$ K${}^{-1}$) corresponding to the mentioned compositions at T=300 K, T=400 K and T=500 K, for a $Si_{c}Ge_{1-c}$ wire of length L=3 mm.

Temperature	$\lambda_{\mathrm{Si}}$	$\lambda_{Ge}$
T=300K	149.95	77.95
T=400K	113.54	59.42
T=500K	92.01	48.08

**Table 3:** Values of $\lambda_{opt}$ (in W m${}^{-1}$ K${}^{-1}$) for the compositions $c_{opt}$ at T=300K, T=400K and T=500K, for L=3 mm.

Temperature(K)	$c_{opt}$	$\lambda_{opt}$ (in W m${}^{-1}$ K${}^{-1}$)
T=300	0.385989	7.51235
T=400	0.375079	7.48291
T=500	0.36537	7.42273
